# Upfront treatment with the first and second-generation tyrosine kinase inhibitors in Ph-positive acute lymphoblastic leukemia

**DOI:** 10.18632/oncotarget.22206

**Published:** 2017-10-31

**Authors:** Guopan Yu, Fang Chen, Changxin Yin, Qifa Liu, Jing Sun, Li Xuan, Zhiping Fan, Qiang Wang, Xiaoli Liu, Qianli Jiang, Dan Xu

**Affiliations:** ^1^ Department of Hematology, Nanfang Hospital, Southern Medical University, Guangzhou, China

**Keywords:** tyrosine kinase inhibitor, BCR/ABL, acute lymphoblastic leukemia, efficacy, safety

## Abstract

The treatment of Ph-positive acute lymphoblastic leukemia (Ph+ ALL) has entranced tyrosine kinase inhibitors (TKIs) era. Currently both imatinib and dasatinib are registered as the front-line treatment for Ph+ ALL, and the other 2^nd^-generation TKIs are suggested as an alternative for those who failed the first-line treatment. However, it remains unclear who could benefit from the 2^nd^-generation TKIs as the first-line treatment for Ph+ ALL. In this study we compared the efficacy and safety of the 1^st^ and 2^nd^-generation TKIs in the front-line treatment of Ph+ ALL and found a trend toward better disease-free survival (DFS) in the 2^nd^-generation TKIs group, though no significant difference in early response and long-term survival between the two groups. Furthermore, subgroup analysis showed that if allogeneic hematopoietic stem cell transplantation (allo-HSCT) was incorporated as consolidation, the 2^nd^-generation TKIs benefited patients with better DFS and overall survival (OS). The two generation TKIs were well tolerated. Higher incidence of acquiring T315I mutation was observed in the patients relapsed on the 2^nd^-generation TKIs. These findings suggested front-line treatment of Ph+ ALL with the 2^nd^-generation TKIs might benefit patients with better survival when allo-HSCT was incorporated as consolidation therapy; meanwhile, the higher incidence of T315I mutation in patients relapsed on the 2^nd^-generation TKIs deserved further attention.

## INTRODUCTION

The Philadelphia chromosome (Ph), resulting from fusion of *BCR-ABL* gene, is the most common cytogenetic abnormality in adult patients with acute lymphoblastic leukemia (ALL), occurring in about 20% to 30% of all cases [1–2]. The incidence increases with age, accounting for up to 50% of patients above the age of 50 [3]. Before the introduction of tyrosine kinase inhibitors (TKI), the outcome of the majority of patients with Ph-positive (Ph+) ALL was poor [4–6]. Combining the first (1^st^)-generation TKI imatinib with chemotherapy with or without allogeneic hematopoietic stem cell transplantation (HSCT) has substantially improved the survival of Ph+ ALL patients [7–10], with longtime overall survival (OS) ranging from 40%–65%, compared to the 20%–40% in pre-imatinib era. Therefore it has become the standard of care. The second (2^nd^)-generation TKIs, for example dasatinib, is 300-fold more active than imatinib *in vitro* [11–12], and has shown marked efficacy in relapsed patients or those refractory to imatinib [13–15], especially in those with imatinib-resistant BCR-ABL mutations. More recently, the 2^nd^-generation TKIs have been used as first-line treatment with promising results [16–19]. Thus we wonder whether the 2^nd^-generation TKIs could replace the 1^st^-generation TKI as the upfront treatment for Ph+ ALL. However, up until now, to our knowledge, there were no randomized trials directly comparing the 1^st^ and 2^nd^ -generation TKIs in treating newly diagnosed Ph+ ALL [20]. Literature review of prospective studies of each TKIs showed no evidence that the 2^nd^-generation TKIs provided better OS [9-10, 16-19, 21]. Furthermore, patients who were treated with dasatinib had higher incidence of pleural effusions and hemorrhage, and there was more concern of developing T315I mutation [16-17, 22-23]. Currently both imatinib and dasatinib are registered as the front-line treatment for Ph+ ALL, and the other 2^nd^-generation TKIs are suggested as an alternative to those who failed the first-line treatment [20, 24]. Whether the 2^nd^-generation TKIs could replace imatinib and provide better outcome when being used as the first-line treatment for Ph+ ALL need further clinical study. This single center study has focused on the efficacy comparing the 1^st^ and 2^nd^-generation TKIs in the front-line treatment of Ph+ ALL.

## RESULTS

### Characteristics of patients

Of all the 109 newly diagnosed Ph+ ALL patients, only 77 patients were given upfront treatment with TKIs and enrolled in this study. Patients were grouped based on the TKIs they received, first vs second generation: 45 on imatinib (43 at the dose of 400 mg daily), 30 on dasatinib (20/30 at the dose of 100 mg daily) and 2 on nilotinib. Fifty-three of 77 patients received allo-HSCT.

We had a male dominant patient population with male to female ratio 52 to 25. The median age of onset was 30 (13-59), and the median peripheral WBC was 61.7(0.7-517.0) ×10^9^/L. Thirteen percent (10 out of 77) cases behaved as Biphenotypic Acute Leukemia (BAL) according to European Group for the Immunological Characterization of Leukemia (EGIL) classification [25]. Fifty one patients (66%) harbored BCR-ABL1-p190 and 26 (33.8%) carried BCR-ABL1-p210; 30 (39.0%) had additional chromosomal abnormalities (ACAs), and 6 (17.1%) presented with ABL1 mutations. The demographic characteristics were comparable between the groups treated with the 1^st^- and with 2^nd^-generation TKIs, with exception of higher carrier of p190 in patients receiving the 1^st^-generation TKI. Details of patient characteristics were listed in Table 1.

**Table 1:** Clinical characteristic of 77 patients with Ph+ ALL

	Total	1^st^-generation TKI	2^nd^-generation TKIs	P-value
Patients (N)	77	45	32	
Median age (years) (range)	30 (13-59)	28 (13-59)	32 (14-59)	0.135
Gender, male/female (ratio)	52/25 (2.1)	28/17(1.6)	24/8 (3.0)	0.238
WBC count (×10^9^/L)	61.7(0.7-517.0)	77.5 (0.7-352.0)	40.2 (1.0-517.0)	0.812
EGIL classification: ALL/BAL	67/10	40/5	27/5	0.561
ACAs (yes/no)	47/30	25/20	22/10	0.242
BCR/ABL1- P190/P210	51/26	34/11	17/15	0.040
ABL1 gene mutations (yes/no) (n=35)^*^	29/6	20/2	9/4	0.100
Allo-HSCT (yes/no)	53/24	34/11	19/13	0.131

Abbreviations: TKI, tyrosine kinase inhibitor; WBC, white blood cell; EGIL, European Group for the immunological classification of leukemias; ACA, additional chromosomal abnormality; Allo-HSCT, allogeneic hematopoietic stem cell transplantation.

^*^35 patients were available for ABL1 mutation detection. Among the 6 patients with ABL1 gene mutations, no T315I mutation was found.

### Early treatment responses

**Figure 1:**
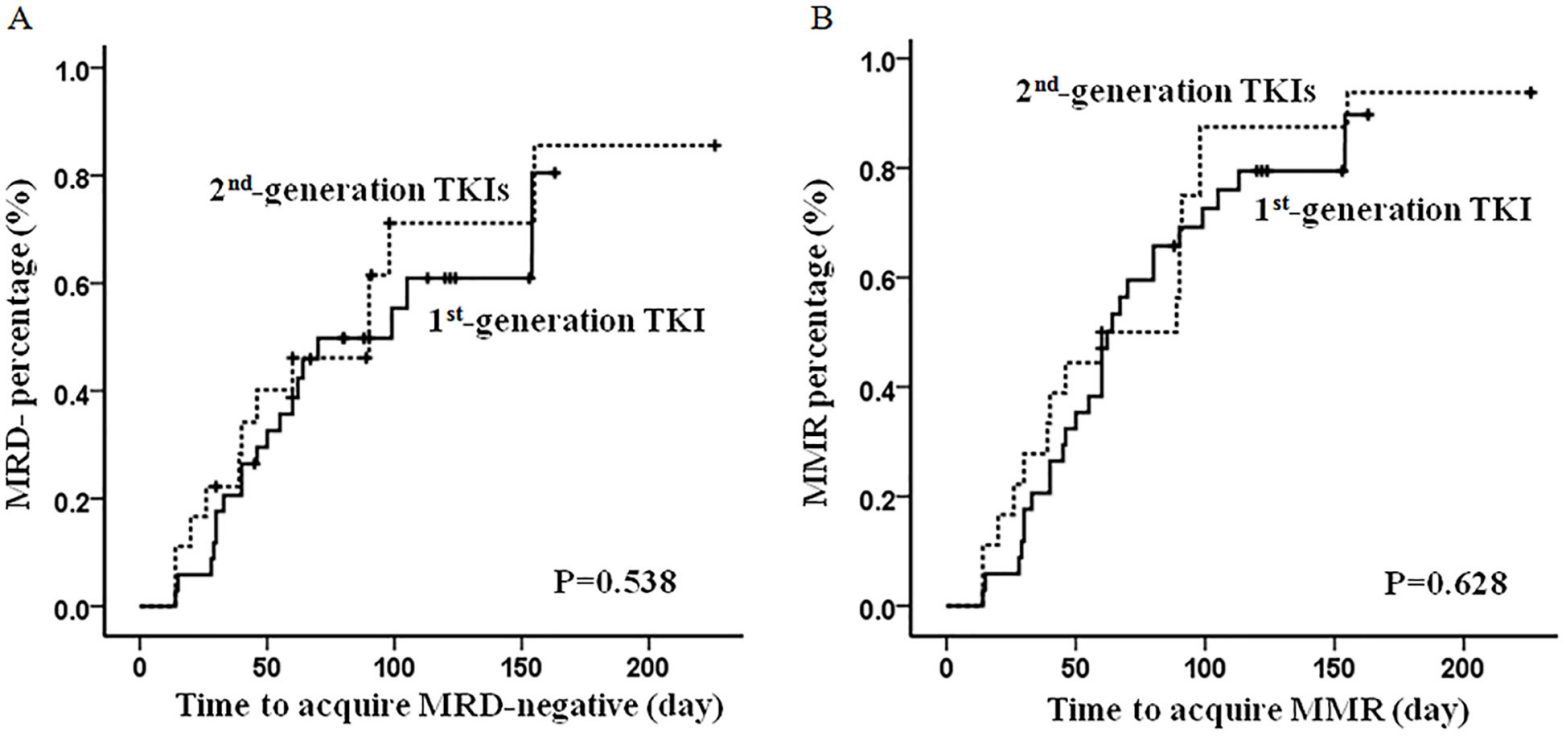
The median time to achieve MMR and MRD-negative No significant difference in the median time to achieve MRD-negative **(A)** and MMR **(B)** were observed between the patients received the 1^st^- and 2^nd^-generation TKIs as front-line treatment.

After the first cycle of induction therapy, 56 (72.7%) patients achieved CR, and 22 (28.6%) had MMR. The numbers of patients having CR and MMR following two cycles of induction therapy increased to 65 (84.4%) and 47 (61%) respectively. Accumulatively there were 72 (93.5%) patients having CR and 32 (41.6%) being MRD negative prior to allo-HSCT. No statistic difference were observed in patients being treated with the 1^st^-generation TKI compared to those treated with the 2^nd^-generation TKIs, with respect to early response following induction therapy, total CR/MMR rates, median time to CR/MMR, and percentage of MRD status (Figure 1); Nor did we observe any difference in early relapse, defined as disease relapse prior to SCT (Table 2). We further performed univariate and multivariate analysis to identify risk factors, including gender, age of onset, WBC count, immunology, chromosome abnormalities, BCR/ABL1 subtype, ABL1 mutation, and TKI that patients received, which could potentially impact patients’ early response. Both BCR-ABL1-p210 transcript and ABL1 mutations were the risk factors adversely affecting patients’ early molecular response (Table 3).

**Table 2:** The treatment responses of the patients with Ph+ ALL

	Total	1^st^-generation TKI	2^nd^-generation TKI	P-value
Patients (N)	77	45	32	
1^st^ cycle CR rate (%)	56 (72.7)	32 (75.0)	24 (71.1)	0.706
MMR rate after 1^st^ induction	22 (28.6)	15 (33.3)	7 (21.5)	0.273
2^nd^ cycle cumulative CR rate	65 (84.4)	39 (86.7)	25 (81.3)	0.518
Cumulative MMR rate after 2^nd^ chemotherapy	47 (61.0)	29 (64.4)	18 (56.3)	0.467
Total CR rate before SCT	72 (93.5)	43 (95.6)	29 (90.6)	0.387
Median time to CR (days)	33.5 (11-113))	40 (11-113)	27 (11-80)	0.103
MRD negative rate before SCT	32 (41.6)	20 (44.4)	12 (37.5)	0.542
Time to MRD negative (days)	60 (14-226)	61 (14-163)	60 (14-226)	0.623
Relapse before SCT	22/72 (30.6)	13/43 (30.2)	9/29 (31.0)	0.942
TTP before SCT (days)	135 (22-450)	114 (22-450)	201 (22-310)	0.471
DFS (days)	263 (22-2134)	247 (22-2134)	280 (22-1094))	0.890
OS (days)	456 (59-2327)	478 (71-2327)	425 (59-1221)	0.264
CNSL	20 (26.0)	12 (26.7)	8 (25.0)	0.869
T315 mutation/relapsed cases^*^	5/17 (29.4)	1/9 (11.1)	4/8 (50.0)	0.079

Abbreviations: CR, complete remission; MMR, major molecular response; MRD, minimal residual disease; TTP, time to progress; DFS, disease free survival; OS, overall survival; CNSL, center nervous system leukemia.

^*^17 out of 33 relapsed patients were available for ABL1 gene mutation detection.

**Table 3:** The adverse factors for early treatment response

	1^st^ cycle-MMR			2^nd^ cycle cumulate-MMR			MRD-negative		
	HR	95% CI	P value	HR	95% CI	P value	HR	95% CI	P value
Univariate factors ^a^									
Gender (male vs. female)	1.69	0.60-4.73	0..319	1.20	0.45-3.23	0.712	0.96	0.37-2.49	0.924
Age (≥35y vs. <35y)	2.10	0.67-6.54	0.200	1.24	0.47-3.24	0.667	1.16	0.44-3.04	0.763
WBC (≥1000×10^9^/l vs.<100×10^9^/l)	1.66	0.58-4.71	0.342	2.44	0.95-6.27	0.064	1.22	0.48-3.08	0.677
EGIL classification (BAL vs. ALL)	4.11	0.49-34.56	0.193	1.68	0.44-6.38	0.446	0.68	0.18-2.56	0.563
ACA (with vs. without)	1.12	0.41-3.08	0.825	0.74	0.29-1.89	0.530	1.41	0.56-3.56	0.468
BCR/ABL1 (P210 vs. P190)	7.74	1.65-36.41	0.010	2.55	0.97-6.74	0.059	3.47	1.20-10.05	0.022
ABL1 gene mutations (yes vs. no)	2.75E8	2.75E8	<0.001	1.13E8	1.13E8	<0.001	4.73E7	4.73E7	<0.001
The generation of TKI (2^nd^ vs. 1^st^)	1.79	0.63-5.06	0.276	1.41	0.56-3.56	0.468	1.33	0.53-3.37	0.543
Multivariate factors^b^									
BCR/ABL1 (P210 vs. P190)	7.99	1.53-41.86	0.014				4.80	1.38-16.77	0.014
ABL1 gene mutations (yes vs. no)	4.86E7	4.86E7	<0.001	2.31E8	2.31E8	<0.001	6.92E7	6.92E7	<0.001

Abbreviations: HR, hazard ratio; 95%CI, 95% confidence interval

^a^ Factors with P<0.10 in the univariate analyses were subjected to multivariate analysis afterwards.

^b^ Backward stepwise Cox proportional-hazard modeling was used in multivariate analysis of risk factors.

### DFS and OS

**Figure 2:**
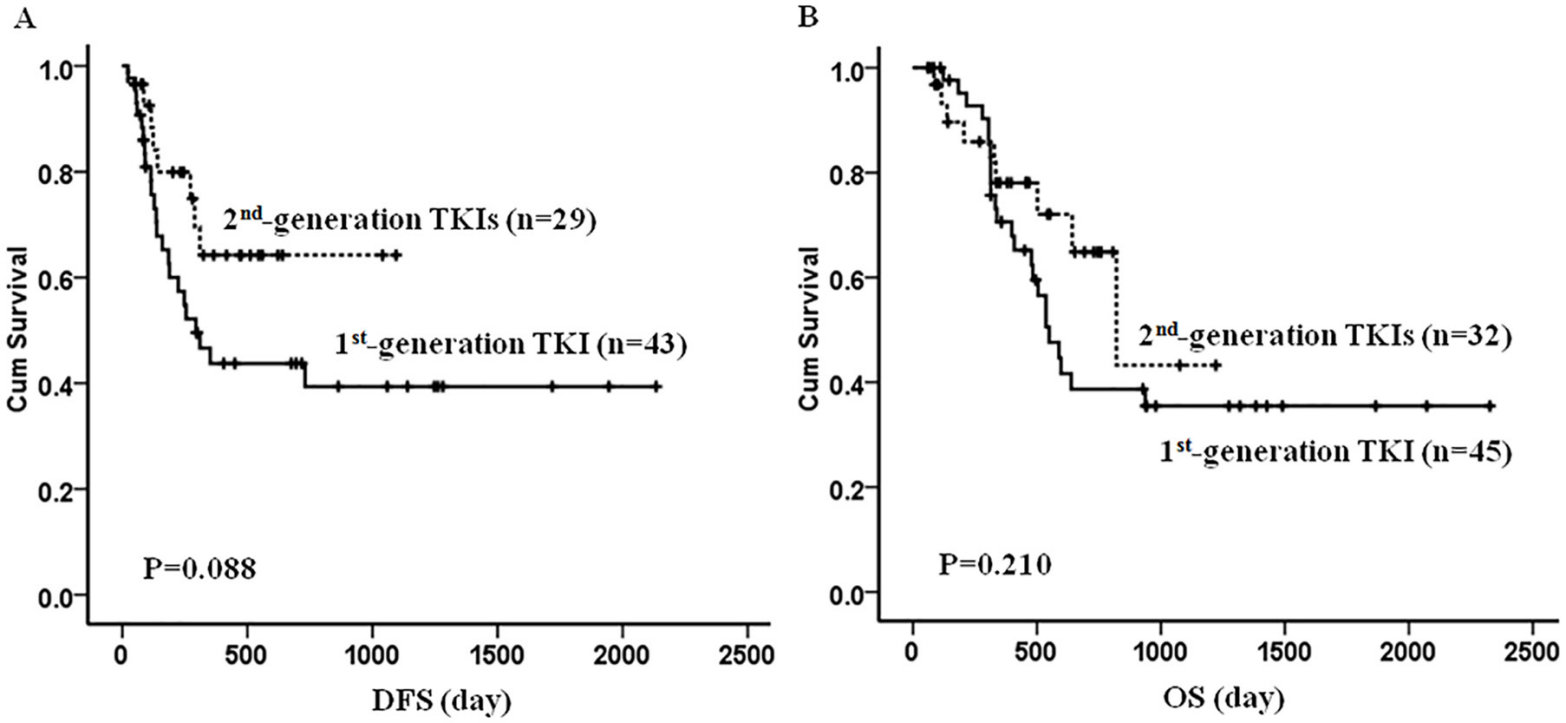
The DFS and OS in the patients front-line treated with the 1^st^- and 2^nd^-generation TKIs An apparently, though not significantly, better DFS was observed in patients being treated with the 2^nd^-generation TKIs compared with that in the 1^st^-generation TKI group (**A**, *P=* 0.088). The OS was comparable between the two groups (**B**, *P=*0.210).

**Figure 3:**
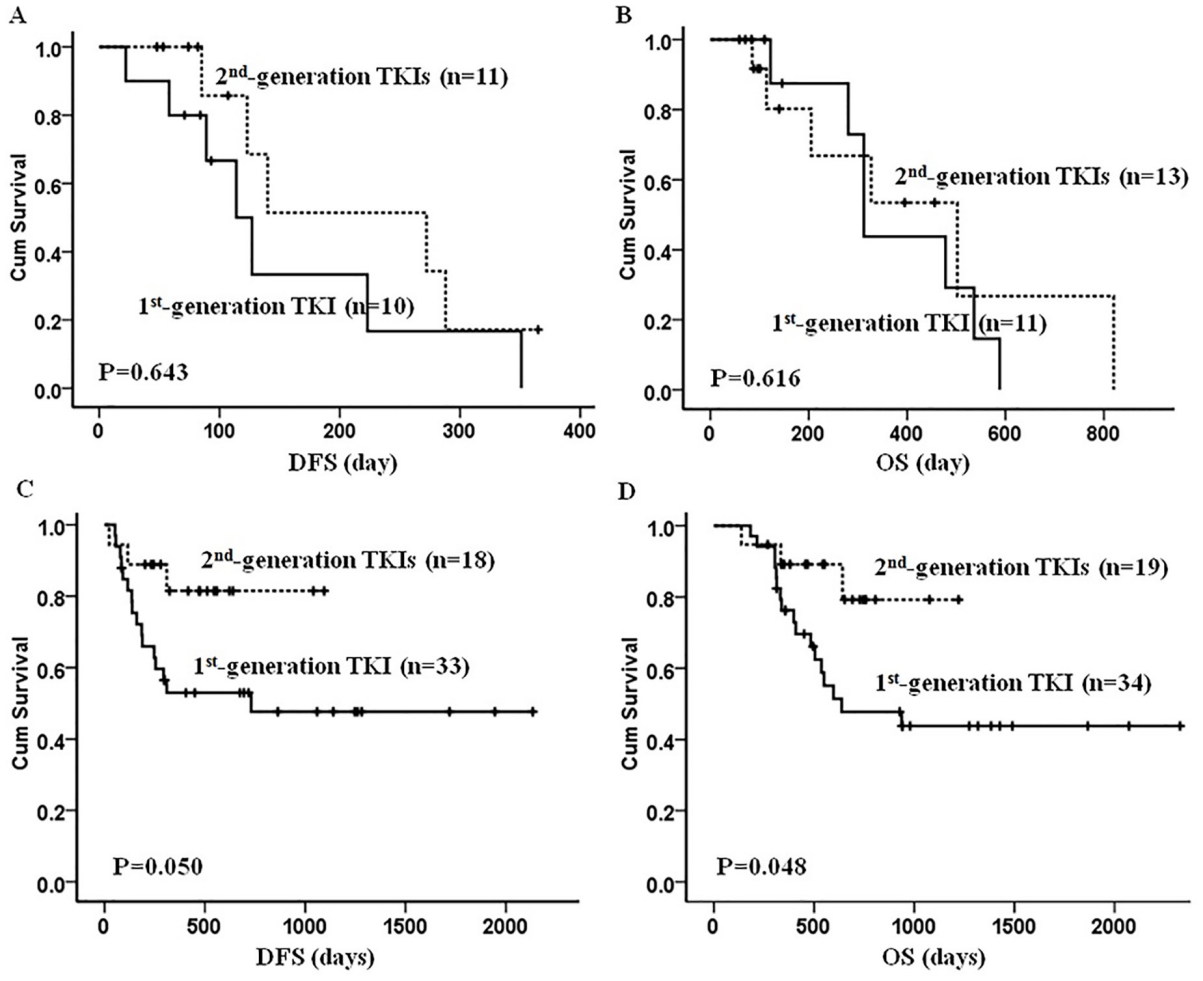
The DFS and OS in the patients front-line treated with the 1^st^- and 2^nd^-generation TKIs combining with or without allo-HSCT Figure **(A, B)** showed that among the patients without receiving allo-HSCT, there was no obvious difference in the DFS between the two groups (DFS, P=0.643; OS, P=0.616). Figure **(C, D)** showed that among the patients receiving allo-HSCT, both DSF and OS were significantly better in the 2^nd^-generation TKIs group compared with that in the 1^st^-generation TKI group (DFS, P=0.050; OS, P=0.048).

With median follow-up of 456 (59-2327) days, 33 (33/72, 45.8%) patients relapsed and 22 relapsed prior to SCT. A total of 33 (33/77, 42.9%) patients died: 25 from relapsed or refractory disease and 8 from treatment related events. Compared to patients receiving the 1^st^-generation TKI, we noted a trend toward better DFS in patients being treated with the 2^nd^-generation TKIs (*P=* 0.088), although this difference was not statistically significant. The OS was comparable between the two groups (*P=*0.210) (Figure 2). As expected, patients undergoing allo-HSCT had much longer DFS (1615±144 vs. 192±32 days, P<0.001) and OS (1463±147 vs. 402±57 days, P<0.001) than those without allo-HSCT. Interestingly, if patients underwent allo-HSCT, there was better survival in both DFS (*P*=0.050) and OS (*P*=0.048) when patients were given upfront therapy with the 2^nd^-generation TKIs compared to those on the 1^st^-generation TKIs. We did not, however, note any difference in survival in non-allo-HSCT patients regardless of TKIs they received (Figure 3). The univariate and multivariate analysis further suggested that upfront treatment with the 2^nd^-generation TKIs could improve long term survival (Table 4).

**Table 4:** The risk factors for DFS and OS in ph-ALL

	DFS			OS		
	HR	95% CI	P value	HR	95% CI	P value
Univariate factors ^a^						
Gender (male vs. female)	1.99	0.76-5.25	0. 163	0.91	0.35-2.39	0.851
Age (≥35y vs. <35y)	1.30	0. 58-2.92	0. 523	2.50	1.01-6.18	0. 048
WBC (≥1000×10^9^/l vs.<100×10^9^/l)	1.17	0.51-2.67	0. 712	1.00	1.00-1.01	0. 279
EGIL classification ( BAL vs. ALL)	1.48	0.43-5.09	0. 538	2.80	0.72-10.83	0. 136
ACA (with vs. without)	0.81	0.37-1.78	0. 598	1.20	0.50-2.90	0. 687
BCR/ABL1 (P210 vs. P190)	0.65	0.24-1.76	0. 394	0.60	0.21-1.75	0.352
ABL1 gene mutations (yes vs. no)	0.82	0.27-2.48	0.727	0.82	0.31-2.20	0. 697
The generation of TKI (2^nd^ vs. 1^st^)	0.34	0.12-0.92	0. 034	0.42	0.42-3.56	0.069
Allo-HSCT (with vs. without)	0.31	0.13-0.75	0.010	0.38	0.16-0.93	0.034
Multivariate factors^c^						
The generation of TKI (2^nd^ vs. 1^st^)	0.40	0.18-0.91	0.030	0.41	0.18-0.93	0.033
Allo-HSCT (with vs. without)	0.23	0.10-0.49	<0.001	0.24	0.11-0.51	<0.001

### ABL1 gene mutations

Six out of 35 (17.1%) patients had ABL1 mutations at diagnosis. The outcomes of these patients were: one maintained CCR, one had refractory disease and could not attain CR, and the rest four relapsed including two undergoing allo-HSCT (Table 5). Among 33 patients who relapsed, 17 were available for ABL1 mutation examination, and 7 (7/17, 41.2%) harbored mutant genes, including 5 with T315I mutation (Table 5). According to the previous reports [16, 18, 23], T315I mutation could be induced or positively selected by the 2^nd^-generation TKIs, and mainly contributed to leukemia relapse, we further did subgroup analysis of the frequency of T315I mutation in the patients relapsed on the 1^st^ vs. 2^nd^-generation TKIs, and found that the incidence in the 2^nd^-generation TKI group was apparently, though not statistically, might be due to small case number, higher than that in the 1^st^-generation TKI group (4/8 vs. 1/9, P=0.079) (Table 2).

**Table 5:** ABL1 gene mutations at diagnosis and relapse in ph+ ALL

Patients	TKI for front-line treatment	ABL1 mutations at diagnosis	Status	ABL1 mutations at relapse
Case 1	dasatinib	F317L	Relapse	No mutation
Case 2	dasatinib	L248V	Relapse	Y253H, E459K
Case 3	imatinib	L523P, E505K	CCR	
Case 4	nilotinib	No mutation	Relapse	T315I
Case 5	imatinib	F317L	NR	
Case 6	dasatinib	No mutation	Relapse	T315I
Case 7	nilotinib	Y253	Relapse	Y253, E255K
Case 8	dasatinib	c.944C>T/p	Relapse	T315I
Case 9	dasatinib	No mutation	Relapse	T315I
Case 10	imatinib	No mutation	Relapse	T315I

### Toxicity

All patients tolerated TKIs well, with exception of 3 patients from the 2^nd^-generation TKI group and 5 from the 1^st^-generation TKI group whose treatment were temporally on hold because of severe bone marrow suppression. One patient with dasatinib had subcutaneous hemorrhage not attributable to platelet count or coagulation status. Pleural effusion was observed in patients receiving both the 1^st^ (3 out of 45 patients) and 2^nd^ (5 out of 32 patients) generation TKIs (P>0.05)

## DISCUSSION

TKI combined with multiagent chemotherapy has been well accepted as the front-line treatment of Ph+ ALL, and the patients under 65 are recommended to receive allo-HSCT if a matched donor is available [20, 24]. In this study, we confirmed the survival advantage of consolidation with allo-HSCT in Ph+ ALL patients, even though TKIs had been continuously used through induction and consolidation. More importantly, our study indicated that the survival benefit from upfront treatment with the 2^nd^-generation TKI only occurred in patients who received allo-HSCT but not in those who were treated with chemotherapy alone.

In the past the significant survival advantage of incorporation of imatinib into the treatment of Ph+ ALL patients has established imatinib as the standard of care [7–10]. However, there is concern of acquired or intrinsic resistance to imatinib, mainly due to BCR-ABL mutation or over-expression and the activation of Scr kinase pathway, leading to treatment failure [26–28]. The 2^nd^-generation inhibitors, in particular dasatinib, were reported to be capable of overcoming such resistance and have higher antileukemic efficacy. It has been widely used to treat patients who are refractory to or untolerate to imatinib, and has shown promising result [13–15]. More recently, the 2^nd^-generation TKIs have been used as the first-line therapy in newly diagnosed Ph+ ALL patients. A report of 53 adults with newly diagnosed Ph+ ALL received combination of dasatinib with prednisone for induction and free post-induction therapy showed that all evaluable patients attained complete hematologic remission (CHR), and the 20-month OS and DFS were 69.2% and 51.1 %, respectively [16]. A phase 2 study [17] of dasatinib with hyper-CVAD for the front-line treatment was conducted in 35 patients with newly diagnosed Ph+ ALL and reported a CR rate of 94 % and the estimated 2-year OS rate of 64 %. The recent follow-up update reported on 72 patients and showed a CR rate of 96 %, with a median DFS and OS of 31 and 47 months [18]. Thus, as imatinib, dasatinib has been recommended as the front-line treatment for newly diagnosed Ph+ ALL [20, 24]. These prospective non-randomized controlled studies, however, showed no evidence that the dasatinib provides survival benefit to Ph+ ALL patients when compared with imatinib. Only some patients in those trials received allo-HSCT. Up until now, there is no study directly comparing the treatment of the two generations TKIs in patients undergoing allo-HSCT.

In our study, among the 53 patients consolidated with allo-HSCT, 34 were front-line treated with imatinib, 19 received the 2^nd^-generation TKIs, mainly dasatinib. With median follow-up of 456 (59-2327) days, there was no significant difference in DFS and OS between the two groups. Subgroup analysis, however, showed benefit in DFS (p=0.05) and OS (p=0.048) in patients treated with the 2^nd^-generation TKIs only when they underwent allo-HSCT. The benefit from TIKs disappeared if patients were treated with chemotherapy only. It is well known that the frequency of T315I mutation and/or multiple ABL1 mutations are higher in patients with long-term TKIs treatment [7, 16-17, 24, 28], which is thought as the major reason for leukemia relapse. Therefore TKIs are only considered as a complement to chemotherapy, serving as a bridge to allo-HSCT. To this date, allo-SCT is still the best treatment option for long term survival in younger and fit Ph+ ALL patients [20, 23–24]. Our result also indicated that among the patients with continuous TKI treatment, those consolidated with allo-HSCT after CR1 had better DFS and OS.

TKI-resistant mutations have been detected in a variable proportion of TKI-naive Ph+ ALL cases [23, 29–30]. Since dasatinib is a dual Src and Abl kinase inhibitor that binds to both active and inactive moieties of the BCR-ABL1 protein and is approximately 300 times more potent against the kinase [11–12], it should be more effective as first-line when incorporated into the treatment. The efficacy of dasatinib was confirmed in the treatment for Chronic Myelogenous Leukemia (CML) [31], although the clinical evidence in Ph+ ALL patients were lacking. In our study, we did not observe significant difference in early response between the two generation TKIs groups; however, the 2^nd^-generation TKIs seemed to reverse the adverse effect of BCR-ABL1-p210 and ABL1 mutation on early molecular response. The presence of TKI resistant mutations, in particular T315I mutation, increased with long term exposure to TKIs, which could explain comparable OS among two groups. If allo-HSCT was available following CR1, the development of TKI-resistance sub-clones were successfully suppressed, and OS was improved.

We confirmed previous clinical trials [13–18] that the 2^nd^-generation TKIs were well tolerated. The main concern is the development of ABL1 mutation, especially T315I mutation. There was 33% to 70% of patients having T315I mutation when relapsed on upfront dasatinib [16–17]; and 65% of patients acquired the T315I mutation when they were rescued with dasatinib after progressed on imatinib [23]. The development of T315I was much higher than that in imatinib failure cases [9, 23]. Similarly, in our study T315I mutation occurred at apparently higher rate in the patients relapsed on the 2^nd^-generation TKIs. Therefore, we need to pay more attention to T315I mutation in the cases failure on the 2^nd^-generation TKI.

To sum up, front-line treatment of newly diagnosed Ph+ ALL patients with the 2^nd^-generation TKIs, especially dasatinib, is as effective and safe as imatinib; when allo-HSCT is incorporated as consolidation therapy following CR1, survival benefit was observed with the 2^nd^-generation TKIs. T315I occurred at higher rate when patients relapsed on the 2^nd^-generation TKIs and deserved further attention. Finally we advise a multicenter prospective control clinical trial to confirm the conclusion.

## MATERIALS AND METHODS

### Patients

One hundred and nine patients were diagnosed as Ph+ ALL, based on the World Health Organization (WHO) 2008 classification, from January, 2010 to January, 2016 in department of Hematology, Nanfang hospital. Seventy seven were given upfront treatment with TKIs combined with chemotherapy with or without allo-HSCT. Informed consents were signed and obtained from all patients or guardians in accordance with the regulations of Nanfang hospital, Southern Medical college of Medicine Institutional Review Boards in agreement with the ’Declaration of Helsinki’.

### Treatments and study plan

TIKs, at the dose of imatinib 400 or 600 mg, dasatinib 100 or 140 mg, or nilotinib 800 mg, by mouth daily, were started at the time of diagnosis and continued throughout the whole treatment. The TIKs were temporarily discontinued when severe bone marrow suppression or severe non-hematologic toxicities were developed. Patients were advised to switch to another TKIs in the setting of relapse. The choice of TKIs was based on patients’ personal preference and economic situations after fully discussing with their physicians. The VI(D)LP (vincristine, daunorubicin or idarubicin, L-asparagines, and prednisone) was given for 1-2 cycles as induction regimen, and this was followed by hyper-CVAD-A (cyclophosphamide, vincristine, daunorubicin, and dexamethasone) alternating with hyper-CVAD-B (high-dose methotrexate and cytarabine) as the consolidation regimen for 2-4 cycles, if CR was obtained; Allo-HSCT were offered if appropriate donors were available. If donors were not available, consolidation regimens with hyper-CVAD-A ± L-asp/B combined with a TKI were continued to 6-8 cycles, and post-consolidation maintenance therapy with a TKI combined with dexamethasone (MTX)/6-Mercaptopurine (6-MP) lasted for 2-3 years. For patients who underwent allo-HSCT, to prevent relapse, reduction of immunosuppressive therapy and prophylactic donor lymphocyte infusion (DLI) were routinely initiated 60-90 days post-transplantation. The use of post-transplant TKIs depended on the MRD status. And TKIs were resumed when BCR/ABL1 transcript increased by one log. All patients received central nervous system leukemia (CNSL) prophylaxis.

If patients relapsed before transplantation, they were suggested to stop and change to another TIKs depending on ABL1 gene mutation. Rescue regimens containing high dose MTX, cyclophosphamide (CTX) or L-Asparaginasum (L-Asp), such as VICLP (VILP plus CTX), CAM (CTX, cytarabine, 6-MP), or high dose MTX plus L-Asp, were given, and allo-HSCT was initiated immediately if a donor was available. If relapse occurred after transplantation, immunosuppression for GVHD was reduced or stopped. DLI was considered if available, and TKIs which were effective in their previous treatment were restarted. If there was concern of leukemia, multi-agent chemotherapy could be initiated.

### Diagnosis of Ph+ ALL and MRD monitoring

Diagnosis of Ph+ ALL was made according to WHO 2008 classification by fluorescence *in situ* hybridization analysis with positive *BCR-ABL1* and/or standard karyotype t(9; 22)(q34; q11). The *BCR-ABL1* fusion transcript, p190 and p210, were detected by using real-time quantitative polymerase chain reaction (RTQ-PCR) (Qiagen, Hilden, Germany) standardized methods with international scale [32]. The MRD, presenting as BCR-ABL1/ABL ratio, detected by RTQ-PCR, was monitored after induction therapy and every consolidation chemotherapy, then at 3-month intervals for the first 2 years of follow up, and at relapse. Major molecular response (MMR) was defined as a BCR-ABL1/ABL ratio of 0.05% in the bone marrow, and molecular CR was defined by the absence of detectable MRD with a sensitivity of at least 0.001% [33].

### ABL1 gene mutations analysis

The samples obtained for RTQ-PCR were also analyzed for the BCR-ABL KD mutation by direct sequencing. Total RNA was extracted from mononuclear cells from the patients’ prophase and relapse samples by TRIzol (Invitrogen), and cDNA was synthesized by reverse transcriptase. Briefly, nested PCR was applied to amplify the complementary DNA region encoding kinase domain of BCR-ABL. All primers were based on the report by Branford et al [34]. Scanning of the ABL KD (amino acids 219-506) for the presence of mutations was sequenced by Sanger [35]. The sample nucleotide sequences were compared to the GenBank accession no. X16416.

### Statistical analysis

SPSS 17.0 software (SPSS Inc., Chicago, IL, USA) was used to evaluate the statistical difference of categorical variables between patient groups with the Pearson Chi-square analysis and Fisher exact test. DFS was calculated from the date of CR to the first relapse or the last follow-up. OS was calculated from the date of diagnosis to the death or the last follow-up. The Kaplan–Meier method and Log rank tests were performed to evaluate DFS and OS between the groups, and a P value of less than 0.05 was considered statistically significant.

## Abbreviations

TKI: tyrosine kinase inhibitor
ALL: acute lymphoblastic leukemia
allo-HSCT: allogeneic hematopoietic stem cell transplantation
MRD: minimal residual disease
CR: complete remission
MMR: major molecular response
DFS: disease-free survival
OS: overall survival
ACA: additional chromosomal abnormality
HR: hazard ratio
95%CI: 95% confidence interval
RTQ-PCR: real-time quantitative polymerase chain reaction
